# Associations between skin barrier characteristics, skin conditions and health of aged nursing home residents: a multi-center prevalence and correlational study

**DOI:** 10.1186/s12877-017-0655-5

**Published:** 2017-11-13

**Authors:** Elisabeth Hahnel, Ulrike Blume-Peytavi, Carina Trojahn, Jan Kottner

**Affiliations:** Department of Dermatology and Allergy, Clinical Research Center for Hair and Skin Science, Charité-Universitätsmedizin Berlin, Charitéplatz 1, 10117 Berlin, Germany

**Keywords:** Dermatology, Nursing homes, Skin conditions, Biophysical measurements, Elderly

## Abstract

**Background:**

Geriatric patients are affected by a range of skin conditions and dermatological diseases, functional limitations and chronic diseases. Skin problems are highly prevalent in elderly populations. Aim of this study was to investigate possible associations between health, functional and cutaneous variables in aged long-term care residents.

**Methods:**

This observational, cross-sectional, descriptive prevalence study was conducted in a random sample of 10 institutional long-term care facilities in Berlin. In total, *n* = 223 residents were included. Demographic and functional characteristics, xerosis cutis, incontinence associated dermatitis, pressure ulcers and skin tears were assessed. Stratum corneum hydration, transepidermal water loss, skin surface pH and skin temperature were measured. Data analysis was descriptive and explorative. To explore possible bivariate associations, a correlation matrix was created. The correlation matrix was also used to detect possible collinearity in the subsequent regression analyses.

**Results:**

Mean age (*n* = 223) was 83.6 years, 67.7% were female. Most residents were affected by xerosis cutis (99.1%; 95% CI: 97.7% - 100.0%). The prevalence of pressure ulcers was 9.0% (95% CI: 5.0% - 13.0%), of incontinence associated dermatitis 35.4% (95% CI: 29.9% - 42.2%) and of skin tears 6.3% (95% CI: 3.2% - 9.5%). Biophysical skin parameters were not associated with overall care dependency, but with age and skin dryness. In general, skin dryness and measured skin barrier parameters were associated between arms and legs indicating similar overall skin characteristics of the residents.

**Conclusion:**

Prevalence of xerosis cutis, pressure ulcers and skin tears were high, indicating the load of these adverse skin conditions in this population. Only few associations of demographic characteristics, skin barrier impairments and the occurrence of dry skin, pressure ulcers, skin tears and incontinence-associated dermatitis have been detected, that might limit the diagnostic value of skin barrier parameters in this population. Overall, the measured skin barrier parameters seem to have limited diagnostic value for the reported skin conditions except xerosis cutis.

**Trial registration:**

This study is registered at https://clinicaltrials.gov/ct2/show/NCT02216526. Registration date: 8th November 2014.

## Background

Geriatric patients are affected by a range of skin conditions and dermatological diseases. Pruritic dry skin (xerosis cutis) is the most common skin disorder in the aged with prevalence ranging from 5.4% to 85.5% [1–3]. In geriatric long-term care settings incontinence-associated dermatitis (IAD), skin tears, and pressure ulcers (PUs) are frequent [4, 5]. The prevalence for IAD was reported to be 22.6%, for skin tears 19.8% [3] and for PUs up to 46% [1]. Intrinsic age-related skin changes include elevated pH, reduced stratum corneum turn-over rates, reduced stratum corneum hydration (SCH) and reduced transepidermal water loss (TEWL) [3, 6, 7]. Extrinsic factors are functional limitations like immobility or incontinence leading to PUs [8] and IAD [9]. Empirical evidence suggests complex relationships between functional decline, age, cognition and the occurrence of adverse skin conditions [10–13]. For instance total Braden scale scores in patients aged 65 + years have been shown to be associated with the development of skin tears [13]. Kilic et al. showed associations between xerosis cutis and being bedridden [14]. An impaired skin barrier may be an indicator for higher susceptibility for skin disorders [3], hence biophysical skin barrier measurements (e.g. TEWL, SCH and pH) are becoming more important for quantifying skin barrier characteristics in geriatric and long-term care research [15, 16]. Aisen et al. proposed that skin hydration is reduced due to immobility in aged patients [17]. However, this finding was never reproduced. Associations and interactions between demographic, functional, clinical skin characteristics and skin barrier properties in geriatric patients have not been investigated systematically yet. We hypothesized that demographic characteristics, skin barrier impairments, and the occurrence of skin diseases in this vulnerable population are interrelated. Therefore, the aim of this study was to investigate possible associations between functional, skin barrier and cutaneous variables in aged long-term care residents and to investigate the strength and directions of these associations.

## Methods

### Study design

This was a descriptive, observational and cross-sectional study. The detailed description of the procedures are provided in the study protocol, which was published previously [18].

### Ethics approval and consent to participate

This study was approved by the ethics committee of the Charité-Universitätsmedizin Berlin (EA1/190/14). Written informed consent was obtained from the residents themselves or their legal representatives prior any study procedure.

### Setting

The study was conducted from September, 30th 2014 to March, 11th 2015 in 10 institutional long-term care facilities in Berlin. Using computer generated random numbers, nursing homes from a list of all existing nursing homes (*n* = 291) were contacted. In case of non-response the next randomly selected nursing home was invited.

### Participants

The inclusion criteria were (1) resident of the respective nursing home, (2) aged ≥65 years, and (3) written informed consent given personally or by legal representative. Residents at the end of life were not considered eligible.

### Variables

Demographic variables like gender and age were collected. The Barthel-Index (BI) was used to measure physical function related to the daily activities with scores ranging from 0 (very care dependent) to 100 (not care dependent) [19]. Skin dryness was assessed with the Overall Dry Skin score (ODS) using a five-point scale ranging from ‘0’ (no skin dryness) to ‘4’ (advanced skin roughness, large scales, inflammation and cracks) [20, 21]. PUs were categorized according to the ICD 10 classification. The Braden scale was used to measure PU risk. Scores range from 6 (high PU risk) to 23 (no PU risk) [22]. IAD was classified according to the IAD-IT classification of Junkin 2008 [23] into four categories: early, moderate, severe, and fungal appearing rush, which may occur in addition to any category of IAD. Skin tears were recorded as present/absent. Cognitive function was measured with the Six Item Cognitive Impairment Test (6-CIT) [24]. Scores range from 0 (no sign of cognitive impairment) to 28 (significant cognitive impairment) [24, 25]. 

Biophysical skin measurements were conducted on intact skin at the right inner midvolar forearm and the right lateral lower leg. TEWL, SCH, pH and temperature were measured using the non-invasive Multi Probe Adapter System MPA® (Courage & Khazaka, Cologne, Germany) with Tewameter®TM 300, Corneometer®CM 825, Skin-pH-Meter®PH 905 and Skin-ThermometerST500. All measurements were performed in triplicates. The arbitrary units (a.u.) for SCH measurement range from 0 to 120, whereas higher readings indicate higher SCH. Elevated TEWL values indicate an increased evaporation of water molecules from the skin surface. Reference values of human skin pH range from 4 to 6 [26, 27]. Skin surface temperature was measured in C°.

### Data sources and measurement

All participating nursing home residents underwent a demographic, nursing, medical and dermatological examination. Among others, a full skin assessment was conducted by a dermatologist. Based on the possibilities of the institutions (e.g. availability of examination rooms) measurements were standardized as much as possible. However, optimal skin measurement conditions were not always achieved. Therefore, all procedures followed the guidelines for the in vivo measurement of TEWL and SCH in non-clinical settings [20]. The relative humidity (%) and environmental temperature (C°) was monitored throughout all skin measurements. Besides these two factors, the skin surface temperature is one of the most important predictor for TEWL [28]. Therefore, all TEWL estimates were converted to a standardized skin surface temperature of 30 °C according to the method by Mathias et al. [29].

### Bias

Nursing homes were randomly selected from all nursing homes in Berlin to ensure generalizability. All study related procedures and measurements were conducted by trained dermatologists and study assistants according to standard operating procedures. All assessments and measurements were done using previously validated tools.

### Study size

One aim was to measure the prevalence of PUs, IAD, skin tears, dry skin and to estimate skin barrier parameters. It was expected that the point estimates of proportions vary widely. Assuming a prevalence of 0.5 of skin diseases, approximately 280 residents would have been needed to measure this proportion with a desired width of a 95% CI of ±0.06. According to the latest Nursing Care Statistics (2013), the size of the nursing home population in Berlin was approximately 30.000 [30]. Assuming 80 residents per institution and a participation rate of 50% (*n* = 40) it was planned to include seven institutions which results in *n* = 280 (7 x n = 40) cases. All residents of the eligible nursing homes were invited, but participation rate was lower than 50%. In order to reach the planned number, three additional nursing homes were recruited.

### Quantitative variables

The sample was grouped according to gender and care dependency to take possible gender and care dependency differences into account. Care dependency was classified into mild to no dependency (total score 60–100), moderate (total score 20–59) and severe dependency (total score 0–19) based on the BI [31]. PU prevalence was reported for categories I to V and DTI (deep tissue injury) and excluding category I. An ODS of ≥1 was categorized as xerosis cutis, relating to the five-point scale. Residents with sum scores ≥8 according to the 6-CIT were classified as ‘cognitively impaired’ [24].

### Statistical methods

Data analysis was descriptive and explorative. Depending on the level of measurement (nominal, ordinal, continuous), demographic characteristics, clinical scores, skin conditions and skin biophysiological measurements were described using means, medians, proportions, frequencies and associated spread estimates. PU, skin tears, IAD and xerosis cutis prevalence was presented including 95% confidence intervals (CI). All variables were compared between gender and BI categories descriptively. To explore possible bivariate associations, a correlation matrix was created. Depending on level of measurement (e.g. metric and dichotomous) biserial or Pearson correlation coefficients were calculated. A minimum of ≥0.2 or ≤ − 0.2 was considered as a minimum level of association. Based on the strengths and directions of associations and based on biophysiological considerations multivariable logistic and linear regression analyses were conducted. Special emphasis was put on possible associations between skin function, skin condition and skin care. Models were built iteratively to increase model fit indicated by Nagelkerke’s R^2^. The correlation matrix was also used to detect possible collinearity. The Durbin-Watson test and the variance inflation factors (VIF) were calculated to assess possible collinearity. A VIF of >10 and a Durbin-Watson test value <1 or >3 was regarded as indicative for autocorrelation.

## Results

### Participants

Fifty-five nursing homes were contacted. Ten nursing homes agreed to participate. Compared to participating institutions, non-participating were larger in terms of number of beds (mean beds per institution: 104.5 vs. 73.7) privately owned (76% vs. 60%) and non-profit (30% vs. 22%).

**Fig. 1:**
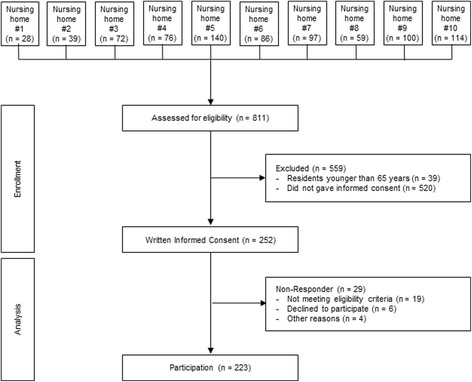
Flow Chart

In total, *n* = 811 nursing home residents lived in the 10 nursing homes at the time of study visits. *N* = 252 residents (31.1%) provided written informed consent and *n* = 223 were included (Fig. 1).

Groups of responders and non-responders of residents had comparable characteristics regarding gender, age and BMI (Table 1), indicating the external validity of the participants.

**Table 1:** Baseline data of responders and non-responders

	Responders (*n* = 223)	Non-Responders (*n* = 29)
Female, n (%)	151/223 (67.7)	16/29 (55.2)
Age [years]		
Mean (SD)	83.6 (8.0)	83.1 (11.1)^a^
Median (IQR)	84 (78–89)	81 (73–95)^a^
BMI [kg/m^2^]		
Mean (SD)	25.3 (5.1)^b^	24.0 (4.4)^c^
Median (IQR)	24.6 (21.9–28.3)^b^	23.5 (20.6–26.8)^c^

*BMI* Body mass index; ^a^ *n* = 223.^b^ *n* = 216.^c^ *n* = 152

### Descriptive data

Sample characteristics are shown in Table 2. Mean age was 83.6 (SD 8.0) years and 67.7% were female. Mean BMI was 25.3 (SD 5.1) kg/m^2^. Mean Braden score was 17.3 (SD 3.7). 77.1% of the residents were cognitively impaired (6-CIT score ≥ 8). The prevalence of xerosis cutis was 99.1% (95% CI 96.8% to 99.8%). Highest mean ODS scores were observed on both lower legs (2.1 (SD 1.0)) followed by the forearms (1.8 (SD 0.9)). Lowest mean ODS scores were graded on the trunk (1.3 (SD 0.8)). PU prevalence was 9.0% (95% CI 5.0% to 13.0%) and 3.6% (95% CI 1.8% to 6.9%) excluding category I. Most of the 20 PUs were located at the sacral region (45%) followed by the heels (25%) in residents with severe care dependency. Four PUs were observed at the back (20%), one at the outer side of the left foot (5%) and one at the plantar side of the foot (5%). No category IV PUs and DTI were observed. IAD was diagnosed in 79 residents (35.4%, 95% CI 29.9% to 42.2%). Most of the residents with IAD were male and had a moderate care dependency. Skin tears were present in 14 residents (6.3%, 95% CI 3.2% to 9.5%) and were mostly located at the arms (80%), followed by the legs (20%). Skin tears on the legs only occurred in residents with severe care dependency. The temperature adjusted mean TEWL was 10.4 (SD 7.2) g/m^2^/h on the midvolar forearm and 8.3 (SD 6.2) g/m^2^/h on the lower leg. Mean SCH was higher on the midvolar forearm (41.2 (SD 9.5) a.u.) than on the lower leg (34.5 (SD 10.2) a.u.). Mean pH values were comparable on both skin areas (5.4 (SD 0.6)). The mean room temperature was 22.9 C° (SD 1.5 C°; IQR 22.0 C° to 24.0 C°) and the mean room humidity was 45.0% (SD 9.2%; IQR 38.0% to 52.0%).

**Table 2:** Demographic characteristics, functional assessments, skin conditions and biophysical skin measurements

	Gender n (%)		Care dependency (Barthel – Index)^d^ n (%)			Total (*n* = 223)
	Female 151 (67.7)	Male 72 (32.3)	Score: 100–60 71 (31.8)	Score: 59–20,116 (52.0)	Score: 19–0 35 (15.7)	
Demographic characteristics						
Age [years]						
Mean (SD)	84.9 (8.0)	80.7 (7.3)	83.0 (7.8)	83.9 (8.1)	83.3 (8.2)	83.6 (8.0)^a^
Median (IQR)	85 (78.0–91.0)	80 (76.0–86.0)	84.0 (77.0–89.0)	85.0 (78.0–90.0)	80.0 (78.0–89.0)	84.0 (78.0–89.0)^a^
BMI [kg/m^2^]						
Mean (SD)	25.5 (5.5)^q^	24.9 (4.3)^k^	25.3 (4.9)^x^	25.6 (5.1)^z^	24.6 (5.5)	25.3 (5.1)^b^
Median (IQR)	25.0 (21.8–28.4)^q^	24.5 (21.8–28.3)^k^	24.7 (21.6–28.7)^x^	25.3 (21.9–28.3)^z^	24.1 (21.7–26.9)	24.6 (21.9–28.3)^b^
Functional assessments						
Braden Total score						
Mean (SD)	17.0 (3.7)^o^	17.9 (3.6)	20.7 (1.8)	16.5 (2.9)	13.0 (3.0)	17.3 (3.7)^d^
Median (IQR)	17.0 (14.0–20.0)^o^	18.5 (15.0–21.0)	21.0 (20.0–22.0)	16.0 (14.3–18.0)	12.0 (12.0–14.0)	18.0 (14.0–21.0)^d^
6-CIT score						
Mean (SD)	21.9 (10.4)	18.4 (11.4)	16.8 (11.9)	21.7 (10.4)	25.6 (6.5)	20.8 (10.8)^a^
Median (IQR)	28.0 (18.0–28.0)	28.0 (6.0–28.0)	25.0 (4.0–28.0)	28.0 (16.5–28.0)	28.0 (28.0–28.0)	28.0 (10.0–28.0)^a^
≥ 8, n (%)	123 (81.5)	51 (70.8)	48 (67.6)	92 (79.3)	33 (94.3)	172 (77.1)^a^
Skin conditions						
Xerosis cutis, Overall Dry Skin Score (arms)						
Mean (SD)	1.7 (0.8)	1.8 (0.9)	1.7 (0.9)	1.8 (0.8)	1.7 (0.9)	1.8 (0.8)
Median (IQR)	2.0 (1.0–2.0)	2.0 (1.0–2.0)	2.0 (1.0–2.0)	2.0 (1.0–2.0)	2.0 (1.0–2.0)	2.0 (1.0–2.0)
Xerosis cutis, Overall Dry Skin Score (legs)						
Mean (SD)	2.1 (1.0)^l^	2.1 (1.0)^m^	2.1 (1.0)^3^	2.1 (1.0)^15^	1.9 (1.0)	2.1 (1.0)^n^
Median (IQR)	2.0 (1.0–3.0)^l^	2.0 (1.5–3.0)^m^	2.0 (1.9–3.0)^3^	2.0 (1.0–3.0)^15^	2.0 (1.0–2.5)	2.0 (1.0–3.0)^n^
Pressure ulcer (I-V/DTI) n, %	14 (9.3)	6 (8.3)	4 (5.6)	9 (7.8)	6 (17.1)	20/223 (9.0)
Pressure ulcer (II-V/DTI) n, %	4 (2.6)	4 (5.6)	1 (1.4)	3 (2.6)	3 (8.6)	8/223 (3.6)
Skin tears (n, %)	8 (5.3)	6 (8.3)	5 (7.0)	6 (5.2)	3 (8.6)	14/223 (6.3)
Incontinence associated dermatitis (n, %)	51 (33.8)	28 (38.9)	24 (33.8)	47 (40.5)	8 (22.9)	79/223 (35.4)
Biophysical skin measurements (Mean, SD)						
Midvolar forearm						
Transepidermal water loss, temperature adjusted, g/m^2^/h	11.3 (8.1)^o^	11.1 (7.6)	11.3 (7.8)	11.9 (9.0)^13^	9.0 (2.2)	10.4 (7.2)
Stratum corneum hydration (a.u.)	41.7 (9.8)^o^	40.1 (8.7)	41.3 (8.8)	41.4 (9.5)^13^	40.1 (10.8)	41.2 (9.5)
Skin surface pH	5.1 (0.6)^o^	5.0 (0.7)^k^	5.1 (0.6)^3^	5.1 (0.6)^13^	5.1 (0.6)	5.1 (0.6)
Skin surface temperature	30.8 (1.3)^p^	31.2 (1.2)	31.2 (1.2)	30.8 (1.3)^14^	30.9 (1.3)	30.9 (1.3)
Lower leg						
Transepidermal water loss, temperature adjusted, g/m^2^/h	8.7 (6.9)^p^	8.8 (7.2)	9.6 (8.7)	8.7 (6.8)^13^	7.1 (2.1)^12^	8.3 (6.2)
Stratum corneum hydration (a.u.)	35.3 (10.2)^o^	32.8 (10.1)	32.5 (9.9)	35.2 (10.6)^13^	36.3 (9.4)	34.5 (10.2)
Skin surface pH	5.4 (0.6)^l^	5.1 (0.6)	5.2 (0.6)^3^	5.4 (0.6)^6^	5.4 (0.6)	5.3 (0.6)
Skin surfaces temperature	30.4 (1.4)^i^	30.8 (1.4)	30.7 (1.2)^3^	30.4 (1.5)^14^	30.5 (1.5)	30.5 (1.4)

*BMI* Body mass index; *DTI* deep tissue injury; *6CIT* 6-item cognitive impairment test; *n.s* not specified; *a.u* arbitrary units.^a^ *n* = 223.^b^ *n* = 216.^c^ *n* = 152.^d^ *n* = 222.^e^ *n* = 63.^f^ *n* = 39.^g^ *n* = 124.^h^ *n* = 60.^i^ *n* = 148.^k^ *n* = 71.^l^ *n* = 147.^m^ *n* = 67.^n^ *n* = 214.^o^ *n* = 150.^p^ *n* = 149.^q^ *n* = 145.^r^ *n* = 50.^s^ *n* = 102.^t^ *n* = 35.^u^ *n* = 77.^v^ *n* = 36.^w^ *n* = 76.^x^ *n* = 69.^z^ *n* = 111.^1^ *n* = 78.^2^ *n* = 26.^3^ *n* = 70.^4^ *n* = 59.^5^ *n* = 40.^6^ *n* = 113.^7^ *n* = 95.^8^ *n* = 47.^9^ *n* = 30.^10^ *n* = 15.^11^ *n* = 18.^12^ *n* = 34.^13^ *n* = 115.^14^ *n* = 114.^15^ *n* = 10

### Main results

The strengths and directions of bivariate associations are shown in Table 3.

Age was most strongly associated with female gender (*r* = 0.285) and SCH on the lower legs (*r* = 0.205). The BI total score was associated with cognitive impairment (*r* = −0.262), the Braden scale total score (*r* = 0.814) and a lower pH on the legs (*r* = −0.204). The Braden total score was also associated with cognitive impairment of the residents (*r* = −0.278) and lower pH on the legs (*r* = −0.222). Intra-individual associations between TEWL, SCH and pH on the midvolar forearm and on the lower legs were high (*r* = 0.800; *r* = 0.427; *r* = 0.574). The skin surface temperature between the forearms and legs were associated as well (*r* = 0.522). A higher ODS on the arm was associated with a lower TEWL (*r* = −0.228) and lower SCH values on the lower legs (*r* = −0.242). Higher ODS on the lower leg was associated with decreased SCH (*r* = −0.281). Skin dryness on the arms and the legs were strongly associated (*r* = 0.614). The occurrence of skin tears were associated with higher TEWL and SCH on the lower legs (*r* = 0.212; *r* = 0.207). PU was associated with decreasing TEWL on the lower legs (*r* = −0.209).

**Table 3:** Correlation matrix (bold marking indicate *r* ≥ 0.2 or ≤ − 0.2)

Variables (mean)	Age	Gender (1/0)	6-CIT score > 8	Barthel Index	Braden score	TEWL arm	TEWL leg	SCH arm	SCH leg	pH arm	pH leg	Skin surface temperature arm	Skin surface temperature leg	ODS arm	ODS leg	IAD	Skin tears(1 = yes/0 = no)	Pressure ulcers (1 = yes/ 0 = no)
Age	1																	
Gender (1 = female/0 = male)	**0.285**	1																
6-CIT score > 8	−0.003	0.110	1															
Barthel Index	−0.085	−0.119	**−0.262**	1														
Braden score	−0.038	−0.115	**−0.278**	**0.814**	1													
TEWL arm	−0.165	0.034	0.103	0.021	−0.016	1												
TEWL leg	−0.080	0.012	0.079	0.072	0.040	**0.800**	1											
SCH arm	0.143	0.075	−0.057	−0.002	0.012	0.145	0.190	1										
SCH leg	**0.205**	0.112	0.024	−0.145	−0.173	0.065	−0.087	**0.427**	1									
pH arm	0.070	0.101	0.116	−0.024	−0.042	−0.025	0.031	−0.006	0.036	1								
pH leg	0.011	0.194	0.084	**−0.204**	**−0.222**	−0.025	0.007	−0.115	−0.003	**0.574**	1							
Skin surface temperature arm	0.126	−0.134	−0.093	0.044	0.097	−0.036	0.000	0.187	0.120	−0.026	−0.105	1						
Skin surface temperature leg	0.024	−0.125	0.062	0.083	0.094	0.079	0.057	0.061	0.018	−0.098	−0.197	**0.522**	1					
ODS arm	0.168	−0.027	−0.077	−0.016	0.017	**−0.228**	−0.117	−0.152	**−0.242**	0.069	0.016	−0.011	−0.020	1				
ODS leg	0.084	−0.011	−0.143	0.050	0.046	−0.143	−0.086	−0.066	**−0.281**	0.078	−0.012	−0.025	−0.019	**0.614**	1			
IAD	0.035	−0.063	−0.017	0.003	−0.015	0.078	0.137	0.040	−0.009	0.031	−0.029	−0.086	0.024	0.065	−0.001	1		
Skin tears (1 = yes/0 = no)	0.154	−0.059	0.048	−0.056	−0.154	−0.195	**0.212**	0.164	**0.207**	−0.061	−0.038	0.082	0.105	0.097	0.048	0.057	1	
Pressure ulcers (1 = yes/0 = no)	0.064	0.045	0.081	−0.092	−0.089	0.169	**−0.209**	−0.027	0.065	0.086	0.119	0.040	0.069	0.042	0.046	0.072	0.054	1

### Skin barrier parameters

Based on the results of the bivariate associations, linear regression models were created. Results of linear regression models are displayed in Table 4.

A higher age was associated with increasing SCH on the lower legs (β = 0.159; *p* = 0.009) and an increasing SCH on the midvolar forearms (β = 0.352; *p* < 0.001). A lower ODS score (β = − 0.225; *p* = 0.002) and the presence of skin tears (β = − 0.150; *p* = 0.013) was associated with increasing SCH on the lower legs. A higher pH on the lower legs was associated with a higher pH on the midvolar forearms (β = 0.567; p < 0.001). The BI total score and the Braden score had no predictive ability in that model. A higher TEWL on the lower legs was strongly associated with higher TEWL values on the midvolar forearms (β = 0.786; p = <0.001) and decreasing skin dryness on the midvolar forearms (β = −0.122, *p* = 0.003). Higher TEWL on the lower leg as dependent variable shows also a strong association with higher TEWL on the midvolar forearms (β = 0.779, *p* < 0.001). The presence of skin tears and PU shows no predictive ability.

### Clinical outcomes

Results of linear regression models, based on the results of the correlation matrix (Table 3) are displayed in Table 5.

Increasing skin dryness on the arms was associated with decreasing TEWL on the midvolar forearms (β = −0.203; *p* = 0.002) and SCH on the lower legs (β = −0.230; p = < 0.001). Increasing skin dryness on the lower legs was associated with decreasing SCH on the lower legs (β = −0.143; *p* = 0.010) and increasing skin dryness on the midvolar forearms (β = 0.576; *p* < 0.001).

**Table 4:** Linear regression dependent variables: Skin barrier parameters

Independent variables	Dependent variable: SCH leg (mean)		
	Standardized Beta coefficient (95% CI)	*P* value	VIF
Demography			
Age (years)	0.159 (0.052 to 0.352)	0.009	1.1
Skin physiology			
SCH (arm)	0.352 (0.255 to 0.514)	<0.001	1.1
ODS (arm)	−0.098 (−2.968 to 0.611)	0.195	1.7
ODS (leg)	−0.225 (−3.940 to −0.859)	0.002	1.6
Skin tears	0.150 (1.336 to 11.095)	0.013	1.1
R^2^	0.304		
Durbin Watson	1.8		
Independent variables	Dependent variable: pH leg (mean)		
	Standardized Beta coefficient (95% CI)	P value	VIF
Demography			
Barthel Index (total score)	−0.088 (−0.007 to 0.002)	0.346	2.9
Braden score	−0.113 (−0.048 to 0.011)	0.228	2.9
Skin physiology			
pH (arm)	0.567 (0.465 to 0.683)	<0.001	1.0
R^2^	0.367		
Durbin Watson	1.8		
Independent variables	Dependent variable: TEWL arm (mean)		
	Standardized Beta coefficient (95% CI)	P value	VIF
Skin physiology			
TEWL (leg)	0.786 (0.813 to 0.995)	<0.001	1.0
ODS (arm)	−0.122 (−1.700 to −0.361)	0.003	1.0
R^2^	0.655		
Durbin Watson	1.9		
Independent variables	Dependent variable: TEWL leg (mean)		
	Standardized Beta coefficient (95% CI)	P value	VIF
Skin physiology			
TEWL (arm)	0.779 (0.605 to 0.749)	<0.001	1.1
Skin tears	0.039 (−1.131 to 3.165)	0.352	1.1
PU	0.076 (−0.109 to 3.462)	0.066	1.0
R^2^	0.648		
Durbin Watson	1.8

**Table 5:** Linear regression, dependent variables: Clinical outcomes

Independent variables	Dependent variable: ODS arm		
	Standardized Beta coefficient (95% CI)	*P* value	VIF
Skin physiology			
TEWL (arm)	−0.203 (−0.039 to −0.009)	0.002	1.0
SCH (leg)	−0.230 (−0.029 to −0.009)	<0.001	1.0
R^2^	0.100		
Durbin Watson	1.2		
Independent variables	Dependent variable ODS leg		
	Standardized Beta coefficient (95% CI)	P value	VIF
Skin physiology			
SCH (leg)	−0.143 (−0.024 to −0.003)	0.010	1.1
ODS (arm)	0.576 (0.529 to 0.776)	<0.001	1.1
R^2^	0.392		
Durbin Watson	1.3		

*VIF* Variance inflation factor

## Discussion

### Key results

This cross-sectional study indicates that nearly every aged nursing home resident is affected by dry skin and more than one third suffered from IAD. In comparison with recently published studies in this setting the prevalences of PUs and skin tears of 9.0% and 6.3% were high [13, 32, 33]. For the first time, the three key skin barrier characteristics TEWL, SCH, and pH were measured in the German nursing home population. Overall, the number of associations was low. Strongest associations have been shown for the TEWL, SCH, pH, skin surface temperature and skin dryness between arms and legs indicating similar overall skin characteristics of the individual residents. Additionally, the functional parameters BI and Braden score were strongly associated with each other as well.

### Interpretation

This was the largest randomly selected sample of nursing homes in which skin barrier measurements and clinical evaluations of different skin conditions have been performed. Study results indicate that nearly every aged nursing home resident is affected by dry skin (99.1%). In comparison, this estimate is much higher compared to reported prevalences in this setting [1, 2]. Leg skin was much drier than arm skin, which is supported by previous studies [2, 34]. Clinical problems caused by dry skin include pruritus, which is also highly prevalent in geriatric patients [34, 35]. Pruritus induces scratching, leading to excoriations and enhances inflammatory reactions leading to secondary infection or superinfection. Ageing related loss of elasticity, dryness, atrophy and laxity of the skin can also lead to an increased skin susceptibility towards infections or skin damages, like PUs or IAD [7, 36]. Additionally, the prevalence of 35.4% of IAD and 8.5% of PU in our sample was also high compared to previously published studies [1, 32]. The low number of associations being detected between biophysical measurements and clinical outcomes in aged long-term care residents indicate that these phenomena seem to be rather independent. Empirical evidence suggests, that there are associations between dry skin and PUs [32]. However, this finding could not be reproduced in this sample. The majority of skin tears was observed at the arms, but there was no association with SCH, pH, TEWL, or skin dryness.

Evidence further suggests that nursing home residents being more care independent are more likely to develop skin tears on the lower legs, whereas residents being more care dependent showed a higher occurrence of skin tears at the arms [10]. This association was not supported by our study results mainly because most skin tears occurred at the arms only. Irrespectively from these findings, recently published studies suggest improvement of xerosis cutis, skin tears as well as IAD by basic skin care interventions and structured skin care regimens [10, 37–39].

More than 77% of the sample was cognitive impaired. Any degree of cognitive impairment, indicated by 6-CIT score > 8, was associated with lower BI and Braden scores. It is well known, that cognitively impaired nursing home residents are more likely affected by urinary incontinence, immobility and PUs compared to cognitively healthy residents [33]. Irrespectively, we were not able to show any associations between skin conditions and care dependency in our sample.

Only few recently published studies examining the associations of skin conditions and skin barrier measurements in nursing home residents exist, which limits the comparability of our results. Age-dependent changes in stratum corneum barrier function, TEWL and pH values are known [15, 40]. The study of Aisen et al. showed a reduced skin hydration in prolonged immobile aged patients [17]. We could not reproduce this finding. Mean TEWL on the midvolar forearms and lower legs was higher compared to previous studies in this population [16, 41], indicating that TEWL is not an absolute value. Changes of TEWL between measurements over the time are more important for interpretation. On the other hand, SCH and pH values were similar to previous reports in this population [16, 42]. In comparison, the pH was higher than in younger age groups [15, 43] indicating a less acidic skin in the elderly, which might affect the regulation of bacterial colonization and the desquamation process of the skin. In our study, lower SCH and TEWL are associated with increased skin dryness, which is in line with physiologically dry skin induced by aging or so called ‘senile xerosis’ [44]. This also indicates that a lower TEWL is certainly not an indicator for a clinical observed improvement in dry skin in this population [38]. Overall, the measured skin barrier parameters seem to have limited diagnostic value for the reported skin conditions except xerosis cutis.

### Limitations

The anticipated sample size of *n* = 280 was not achieved and there were differences between participating and non-participating institutions in terms of size and ownership. Whether this possible selection bias affected the results is unclear. Skin measurements were standardized as much as possible according to the circumstances in the nursing homes (e.g. monitoring of the room temperature and humidity, adjustment of TEWL to the skin surface temperature). All measurements followed the guidelines for the in vivo measurements in non-clinical settings. However, standardized and optimal conditions for the measurements were not achieved. Finally, skin barrier measurements were performed on arm and leg skin areas only. These area specific variables might not be relevant to cutaneous conditions at other skin areas, e.g. IAD.

### Generalizability

Using a population-based approach and randomly selected nursing homes, *n* = 223 aged nursing home residents were included. Facility characteristics were well comparable to all nursing homes in the federal state of Berlin, Germany in terms of sponsorship (privately owned 60% vs. 50.5%; non-profit 30% vs. 45.2%; public 10% vs. 4.2%) or beds per institution (mean 73.7 vs.79.2) [45]. Demographic data like age, gender and care dependency are well comparable with the general German nursing home population statistics (e.g. females 67.7% vs. 72.7%; care-level I: 38.6% vs. 39%; care-level II: 40.8% vs. 40.5%; care-level III 18.4% vs. 21%) [30] which supports the generalizability of the study results. Other characteristics like the mean Braden scale scores were comparable to previous studies in this population and setting, [46–48] indicating external validity of the study results.

## Conclusion

Prevalence of xerosis cutis, PUs and skin tears were high, indicating the load of these adverse skin conditions in this population. Only few associations of demographic characteristics, skin barrier impairments and the occurrence of dry skin, PUs, skin tears and IAD have been detected, that might limit the diagnostic value of skin barrier parameters in this population. In general, the ODS and measured skin barrier parameters are highly associated between arms and legs indicating similar overall individual skin characteristics of the residents.

## Abbrevations

AU: Arbitrary units.
BI: Barthel-Index.
BMI: Body mass index.
CI: Confidence interval.
CIT: Cognitive impairment test.
DTI: Deep tissue injury.
IAD: Incontinence-associated dermatitis.
mL: Mili Litre.
ODS: Overall Dry Skin score.
OR: Odds ratio.
PU: Pressure ulcer.
SCH: Stratum corneum hydration.
SD: Standard deviation.
TEWL: Transepidermal water loss.
VIF: Variance inflation factor.
